# Systematic search for pairwise dependencies of torsion angles

**DOI:** 10.1186/1758-2946-4-S1-P36

**Published:** 2012-05-01

**Authors:** Christin Schärfer, Tanja Schulz-Gasch, Matthias Rarey

**Affiliations:** 1 Center for Bioinformatics (ZBH), Bundesstr. 43, 20146 Hamburg, Germany; 2 F. Hoffmann-La Roche Ltd., 4070 Basel, Switzerland

## 

Most available tools for conformer generation, like OMEGA [1], ROTATE [2], and MIMUMBA [3], divide the conformational space into quantized degrees of freedom, i.e. torsion angles, which are treated independently. The independence of torsions is however not valid for all fragments [4]. There are pairs of mutually dependent degrees of freedom e.g. two consecutive torsion angles in aryl-X-aryl systems. The fact that two torsions are dependent implies that if one of the torsions is set to a specific angle, the set of possible angles for the other torsion is limited. This knowledge could be used to significantly narrow down the conformational space in deterministic rule-based conformation generators.

For our systematic search for pairwise dependent torsion angles, we assembled a set of about 200 chemical patterns, each describing a torsion angle and part of its environment. The patterns range from very general descriptions like 'rotatable bond between two sp3 hybridized atoms' to patterns describing a more specific molecular environment.

As a first approach we tried to replicate the examples given by Bramelt et al. using suitable chemical patterns and a CSD [5] subset of about 73,000 molecules as a database. We then performed a pairwise analysis of all our chemical patterns, including each pattern with itself, using again the CSD subset of about 73,000 molecules. We used two different search scenarios. In our first search the torsion angles had to be directly next to each other while in our second search they had to be exactly one bond apart.

Using our systematic search approach we found many additional examples for dependent torsion angles, confirming the findings of Bramelt et al. and supporting their advice to search for pairs of mutually dependent conformation variables.
